# Digital finance reduces urban carbon footprint pressure in 277 Chinese cities

**DOI:** 10.1038/s41598-024-67315-z

**Published:** 2024-07-17

**Authors:** Zheming Dong, Shujun Yao

**Affiliations:** 1 School of Statistics, Dongbei University of Finance and Economics, Dalian, 116025 China; 2 School of Management, Xi’an University of Finance and Economics, Xi’an, 710100 China

**Keywords:** Digital finance, Urban carbon footprint pressure, Bank branches, Residents' environmental awareness, Sunshine duration, Environmental sciences, Environmental social sciences

## Abstract

As global warming's impact on humanity surpasses initial predictions, numerous countries confront heightened risks associated with escalating urban carbon footprints. Concurrently, digital finance has flourished, propelled by advancements in digital technology. This convergence underscores the urgency of exploring digital finance's role in mitigating urban carbon footprint pressures. This study analyzes data spanning 277 Chinese cities from 2011 to 2020, yielding several key findings: Firstly, we developed a dataset detailing the carbon footprint pressures in these cities, revealing that variations in these pressures predominantly correlate with economic growth. Secondly, our analysis indicates that digital finance has a significant impact on reducing urban carbon footprint pressures, through mechanisms such as reducing the number of physical bank branches and enhancing residents' environmental awareness. Thirdly, the study identifies that the efficacy of digital finance in reducing carbon footprint pressures varies according to factors like sunshine duration and geographic location. The insights from this research aim to contribute substantively to strategies for sustainable urban development.

## Introduction

Since the Industrial Revolution, continuous technological advancements and transformations in industrial production models have propelled large-scale mechanized production. The extensive use of machinery has increased production efficiency but has also escalated energy consumption and the demand for fossil fuels, leading to a rise in carbon emissions^1^. Additionally, sustained economic growth and rapid urban expansion have significantly reduced forest and vegetation areas, continuously diminishing carbon sinks capacities. These changes collectively exacerbate the pressure of urban carbon footprints, highlighting the critical environmental challenges modern cities face^2^. For instance, the International Energy Agency (IEA) reported in its 2022 Carbon Dioxide Emissions Report that China's energy-related emissions were approximately 12.1 billion tons. Although this figure has decreased compared to previous years, it still accounts for 32.88% of global carbon dioxide emissions. In response, the Chinese government has committed under the Paris Agreement to peak carbon emissions by 2030 and achieve carbon neutrality by 2060. Despite considerable efforts, due to rapid economic development and continuous expansion of urban land, the issues of excessive carbon emissions and limited carbon absorption capacities still pose significant negative impacts on the ecological environment^3,4^. Therefore, comprehensively reducing the pressure of urban carbon footprints has become an urgent priority, which is crucial for the sustainable development of both the economy and the ecosystem.

In recent years, numerous studies have begun to explore the goal of reducing carbon footprint pressure by improving factors such as trade, transportation, economics, and population. For example, existing research shows that enhancing the efficiency of transportation modes in import and export trade, as well as encouraging the use of cleaner technologies in trade-related production processes, can reduce urban carbon footprint pressure^5^. At the same time, studies indicate that economic agglomeration can mitigate the rising carbon footprint pressures of urban clusters caused by population growth and increased affluence^6^. Additionally, scholars have discussed how to reduce urban carbon footprint pressure from the perspectives of technological advancement, reducing the use of unsustainable resources, and altering urban structures^7–9^. Notably, these studies share a common gap in knowledge: they overlook the significant contribution of financial instruments in environmental protection. Indeed, financial instruments can link economic consequences with environmental performance, thereby playing a crucial role in reducing urban carbon footprint pressures. However, this aspect has not yet received sufficient attention in the literature.

With the development of the digital economy, digital finance, as a new generation of financial tools, plays a key role in the advancement of markets and technological innovations. Specifically, it contributes to the efficient operation of the entire economic system, enhances innovation capabilities, and facilitates macroeconomic regulation. Particularly, the role of digital finance in environmental sustainability is increasingly prominent^10,11^. Utilizing innovative technologies such as blockchain, artificial intelligence, and cloud computing, digital finance transcends traditional financial boundaries^12,13^, reducing resource waste and promoting green technological innovation. These advancements not only enhance financial inclusivity and literacy^14–16^, but also improve the environmental friendliness of economic activities by enabling cities and businesses to effectively reduce carbon emissions^17,18^. Regrettably, despite the known significant advantages of digital finance in environmental protection, current literature primarily focuses on its emission reduction effects^19,20^, often neglecting its comprehensive role in reducing carbon emissions and enhancing carbon sinks (a key mechanism for absorbing atmospheric carbon dioxide). Similarly, there is currently a lack of a comprehensive perspective to consider the balance between carbon emissions and carbon sinks, and the comprehensive impact of digital finance on this balance has yet to be confirmed.

Therefore, this study utilizes the urban carbon footprint pressure metric to assess the equilibrium between carbon emissions and carbon sinks. Data from 277 cities in China were analyzed through visualization techniques and multivariate linear regression to verify the role of digital finance in mitigating urban carbon footprint pressure. It is hoped that the findings of this research will address existing gaps in knowledge and provide a solid foundation for policymakers in crafting relevant policies. The contributions of this paper include:

- We have integrated carbon emissions and carbon sinks to develop a comprehensive indicator, the urban carbon footprint pressure. Utilizing data from Chinese cities from 2011 to 2020, we calculated its distribution differences and trends, providing theoretical support for the government to assess the effectiveness of environmental policies and to formulate corresponding plans and policies.
- We specifically tested the mitigating effect of digital finance on urban carbon footprint pressure. Moreover, we explored the inhibitory impact of digital finance on urban carbon footprint pressure from aspects such as the degree of digitization, coverage, and depth of use. We aim to address the deficiencies in existing research by conducting a multi-dimensional study, highlighting that the application of digital finance can accelerate and coordinate global emissions reduction efforts.
- Our research delves into how digital finance can reduce urban carbon footprint pressure, primarily through reducing the number of urban bank branches and enhancing residents' environmental awareness. We hope to provide novel insights for policymakers' strategic planning through an in-depth study of the specific impact mechanisms.
- Our heterogeneity analysis reveals that in the eastern regions of China and areas with longer sunlight hours, the alleviating effect of digital finance on urban carbon footprint pressure is more pronounced. These conclusions significantly fill the gaps in current research and aim to provide policymakers and stakeholders with fresh perspectives, offering a robust foundation for global low-carbon development.

## Method

1. *Theoretical analysis* This section offers a theoretical analysis on how digital finance can alleviate pressures on urban carbon footprints. Building on this analysis, the paper presents several hypotheses and conjectures. The analysis is divided into two main aspects: first, the capability of digital finance to mitigate urban carbon footprint pressures; and second, the indirect methods through which digital finance might alleviate these pressures. Based on these insights, we propose the research hypotheses for this study.
2. *Data preparation* In this section, we prepared the data required for our study, which encompasses several key aspects. First, we collected data relevant to this paper, which primarily includes: levels of digital finance in Chinese cities, carbon emission data and carbon sinks data of Chinese cities, GDP of Chinese cities, and foreign investment amounts in Chinese cities. Second, we preprocessed the collected data, removing irrelevant data and filling in missing values using linear interpolation. Third, we calculated the necessary variables for our study, such as urban carbon footprint pressures, per capita GDP, and the degree of dependence on foreign trade.
3. *Model construction* Initially, to investigate the mitigating effect of digital finance on urban carbon footprint pressures, we constructed an econometric model (a two-way fixed effects model). Subsequently, this study incorporates the core explanatory variable (digital finance) along with the interaction terms of mechanism variables into the two-way fixed effects model, aiming to examine the pathways through which digital finance reduces urban carbon footprint pressures. Lastly, we introduced the quadratic term of digital finance into the model to test the nonlinear relationship between digital finance and urban carbon footprint pressures.
4. *Data validation* On one hand, we employed tools such as ArcGIS to visually process the dataset, presenting a clear picture of the distribution and trends in carbon footprint pressures across 277 Chinese cities. Additionally, we explored potential underlying causes. On the other hand, we utilized a linear regression approach (Two-way fixed effects) to confirm the mitigating impact of digital finance in Chinese cities on urban carbon footprint pressures and to elucidate the pathways of this influence.

**Figure 1:**
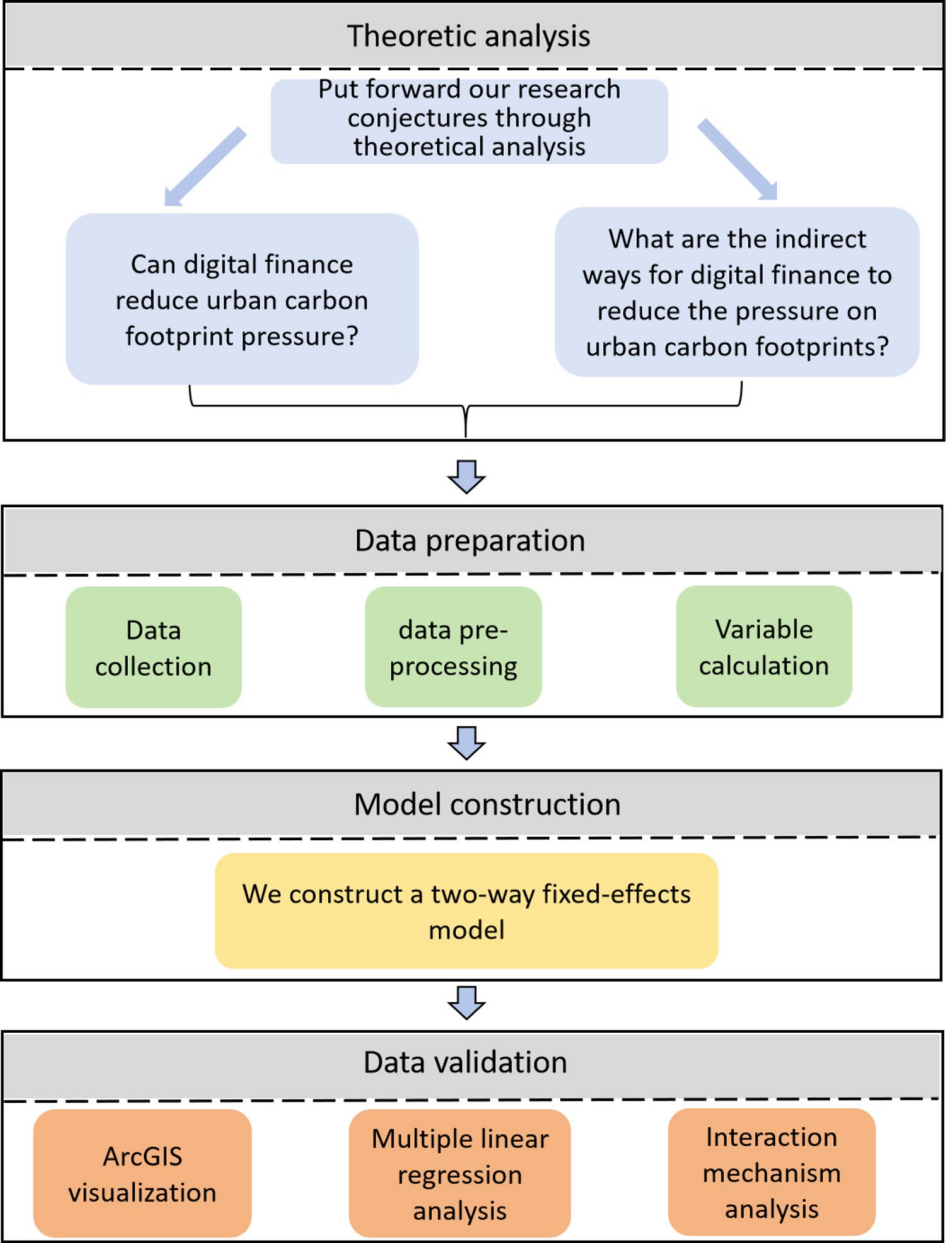
Workfow of the study.

Figure 1 shows more clearly the working method and workflow of this study.

## Theoretic analysis

As global attention to environmental protection and sustainable development intensifies, the study of how financial tools can be utilized to reduce urban carbon footprint pressures has become particularly significant. Based on the theoretical framework provided by the Environmental Kuznets Curve (EKC), it is hypothesized that during the initial stages of economic development, environmental degradation intensifies with economic growth. However, once a nation reaches a certain economic threshold, environmental quality begins to improve with rising incomes. This phenomenon reflects the cumulative impact of economic development, technological progress, and environmental policies. Applying this theory to a comparative study of traditional and digital finance reveals that traditional finance may initially promote rapid industrialization and urbanization, often accompanied by low energy efficiency and high carbon emissions. However, as economic development progresses and environmental awareness increases, society and financial institutions begin to seek greener financial solutions. At this point, the introduction of digital finance marks a new phase in the transition towards higher environmental quality.

Compared to traditional financial mechanisms, digital finance offers significant potential to promote green and sustainable development. The integration of digital finance not only expands the funding channels available to environmental projects but also introduces a higher degree of scrutiny and accountability into the investment process. This shift is crucial for directing funds towards more sustainable urban projects, thereby significantly reducing carbon emissions associated with urban centers. Consequently, the evolution of digital finance may represent a turning point in the economic-environment relationship described by the Environmental Kuznets Curve (EKC), where environmental protection and economic growth begin to coexist harmoniously after reaching a certain level of economic development^21^. Therefore, exploring the transformative potential of digital finance to reduce urban carbon footprints is essential, as it could mark a new phase in aligning economic activities with environmental sustainability.

In practical terms, the impact of digital finance on alleviating urban carbon footprint pressures manifests in two key areas: urban development and corporate production. On one hand, within the realm of urban development, digital finance supports low-carbon transportation and smart city planning. Financial institutions can leverage digital financial tools, supported by government policies, to provide convenient loan and leasing services to car buyers, thereby reducing carbon emissions in urban transport. Additionally, digital financial technologies can support the establishment of digital ecological monitoring systems that enable real-time monitoring and management of urban ecological environments, aiding in the protection and restoration of ecosystems and thereby reducing urban carbon footprint pressures. On the other hand, in corporate production, digital finance facilitates green financing and green insurance, addressing not only funding issues but also emphasizing the importance of considering environmental protection and sustainable development performance in investment decisions. This mandatory and regulatory characteristic compels companies to prioritize green technological innovations to meet investors' and the market’s demand for environmental protection. Consequently, companies achieve low-carbon production through technological green transformation, reducing regional production carbon emissions and thus further alleviating urban carbon footprint pressures. Based on the aforementioned analysis, this paper proposes the following hypothesis:

### Hypothesis 1

Digital finance can effectively reduce urban carbon footprint pressures.

Banks play a crucial role in the financial system, acting not only as intermediaries for funds and payment settlements, but also as drivers of risk management and financial innovation. According to the classical financial intermediary theory^22^, banks facilitate the efficient allocation of resources and stabilize financial markets by transforming funds of varying maturities and addressing issues of asymmetric information. However, the traditional operations of banks are also associated with substantial energy consumption and carbon emissions. The energy consumption of bank branch buildings, lighting, and air conditioning are major sources of carbon emissions^23^. As the number of branches increases, this issue becomes more pronounced. Consequently, traditional banks play a critical role in implementing environmental measures. Yet, in the face of a green transition, traditional banks may encounter dual challenges of motivation and technology. On the one hand, the green transition requires sustained long-term investment with low short-term returns, presenting substantial motivational and practical challenges for some traditional banks at this stage. On the other hand, there is a technological dilemma as there is a significant difference between digital and traditional financial models. Traditional information flows are time-consuming, and the mismatch between costs and benefits necessitates the use of financial technologies such as big data and artificial intelligence to reduce costs and enhance risk control.

Notably, digital finance can play a significant role in reducing the energy consumption and carbon emissions of physical bank branches. With the advancement of digital finance, banks can reduce reliance on physical branches by enhancing the quality and range of online services. Digital services such as online banking and mobile applications not only offer higher efficiency but also significantly lower environmental costs. These measures reduce the necessity for physical branches, thereby decreasing travel frequency and the use of paper, effectively mitigating urban carbon footprints pressure^24^. Furthermore, digital finance allows banks to more accurately analyze and predict market trends, optimizing resource allocation through data-driven decision-making. This increased precision aids banks in investing more effectively in low-carbon technologies and business models, further reducing overall carbon emissions. Based on the analysis above, this paper proposes the following hypothesis:

### Hypothesis 2

Digital finance can reduce urban carbon footprint pressures by decreasing the number of physical bank branches.

Residents, as primary participants in urban activities, have a significant impact on the formation of urban carbon footprints through their environmental awareness and behaviors. According to Norm Activation Theory in social psychology, when residents are highly concerned about environmental issues, their personal behaviors tend to be more eco-friendly. Such behaviors include, but are not limited to, using public transportation to reduce vehicle emissions, optimizing household energy use, choosing renewable energy sources, and actively protecting and expanding local vegetation, all of which theoretically reduce household carbon emissions and enhance carbon sinks.

Digital finance holds unique potential in encouraging residents' eco-friendly behaviors. On one hand, by offering investment opportunities in environmental projects, such as sustainable financial products or green bonds, digital finance platforms can stimulate residents' interest in eco-investments. This approach not only funds environmental projects but also enhances residents' awareness and participation in environmental issues through financial market mechanisms. On the other hand, digital finance can utilize its technological advantages, such as data visualization and real-time information dissemination, to increase residents' awareness of the environmental impacts of their daily activities. For instance, some banks and fintech companies have developed tools and apps that help users track their carbon footprint and provide suggestions for reducing emissions^25^. Furthermore, through reward and incentive mechanisms, digital finance can effectively encourage residents to adopt more eco-friendly consumption and lifestyle choices. For example, banks could launch reward platforms that offer points or discounts to users who opt for electronic billing or use public transportation, significantly boosting their willingness to engage in environmentally friendly actions.

### Hypothesis 3

Digital finance can reduce the pressure on urban carbon footprints by increasing residents' environmental awareness.

## Data preparation

### Data sources

This article uses Chinese cities as a research sample, taking into account the background of digital finance and China’s carbon emission data, and selects 2011–2020 as the time window for the survey sample. Digital financial data comes from the Digital Finance Research Center of Peking University, and other city-related data comes from the "China Statistical Yearbook" and relevant data released by various city governments. Enterprise data comes from the CSMAR database. Additionally, after data collection integration, we removed samples with significant missing values and used linear interpolation to fill in some cities with fewer missing values. After data cleaning, we obtained 10 years of data for 277 cities, forming a sample of 2770 observations.

### Variable calculation

1. *Independent variable* The independent variable in this paper is the Urban Carbon Footprint Pressure (CCF). Urban carbon footprint refers broadly to the pressure of carbon dioxide emissions faced by a city. This pressure arises from both natural and social factors. Prior to measuring carbon footprint pressure, this study reviews existing methods of urban carbon footprint measurement. Moran developed a networked global carbon footprint model, identifying urban–rural consumption patterns and purchasing power as primary predictors of urban residents' carbon footprints^26^. Galli measured regional ecological carbon footprints based on carbon emissions produced by residents' use of natural resources and generation of services^27^. Lombardi provided a phased summary of urban carbon footprint measurement techniques. They highlighted the determination of regional carbon emissions and the definition of 'urban' (or community) as primary considerations^28^. Some scholars argue that if the ratio of carbon emissions to economic output in a city approaches 1, it suggests a balanced development of environment and economy. A ratio exceeding 1 indicates that the city is overburdened with carbon^29,30^. Similarly, urban carbon footprint pressure specifically stems from increased carbon emissions and decreased vegetation absorption capacity. Hence, the measurement of urban carbon footprint pressure should consider both reducing emissions and enhancing carbon sinks. To this end, this study employs Formula (3) to assess urban carbon footprint pressure.
  1 $$ CCF_{i} = CE_{i} /CVS_{i}  $$
  In Formula (1), CCFi represents the Urban Carbon Footprint Pressure; CEi denotes the city's carbon dioxide emission equivalents; and CVSi signifies the carbon absorption capacity of urban vegetation. This method of measuring urban carbon footprints has been widely applied in existing research^31,32^. Urban carbon emission data are calculated according to the "International Standard for Urban Greenhouse Gas Accounting." The steps are as follows: First, establish the accounting boundaries. This paper considers Chinese cities as the accounting units for carbon emissions, using municipal administrative divisions as the basis for accounting boundaries. Second, identify the sources of carbon dioxide emissions. Following the "IPCC Guidelines for National Greenhouse Gas Inventories," greenhouse gas emission sources are categorized into five main types: energy activities, industrial production, agricultural activities, land use changes, and waste disposal. The third step involves data collection and the calculation of urban carbon dioxide emissions using the emission factor method.
  2 $$ GHG_{k} = AD_{k} \times EF_{k} $$
  In Formula (2), GHGk represents the greenhouse gas carbon dioxide (CO2). ADk refers to the activity data for production or consumption activities that cause CO2 emissions, where k represents the activities generating CO2. EFk denotes the emission factor for carbon. Additionally, this study builds on previous research by using field observation methods to collect and calculate data on carbon sinks^33^.
2. *Dependen variable* The dependent variable in this paper is Digital Finance (DIF). In existing research, the most widely used measure of digital finance is the Digital Inclusive Finance Index compiled by the Digital Finance Research Center of Peking University^34,35^. This study selects this index as the basis for measuring digital finance for two reasons: firstly, the Digital Finance Research Center of Peking University collaborates with Ant Financial to organize and compile the data, ensuring its scientific rigor and reliability. Secondly, Ant Financial utilizes internal digital technologies to record comprehensive and objective data on users' electronic transactions. Furthermore, to study the impact of digital finance on urban carbon footprint pressure from multiple dimensions, we utilize the secondary indicators of the digital finance index for multidimensional measurement. These secondary indicators include the degree of digitalization in digital finance (DIG), the breadth of coverage of digital finance (COV), and the depth of usage of digital finance (USE).
3. *Control variables* To reduce the influence of confounding factors such as regional economic development disparities, educational differences, and technological level disparities, and to more accurately study the relationship between digital finance and urban carbon footprint pressure, this study introduces the following control variables: ① Economic development level. ② Foreign trade dependence ③ Educational foundation. ④ Technology level. ⑤ Urbanization degree.
  Table 1 presents the computational explanations and descriptive statistics for the variables in question. According to the descriptive results, the maximum value of urban carbon footprint pressure is 16.51, the minimum value is 0.009, and the mean is 1.363. The mean significantly exceeds the minimum value and is less than one-tenth of the maximum value, indicating substantial disparities in carbon footprint pressure among cities. This further underscores the necessity of this study. The descriptive statistics for other data fall within reasonable ranges, with no evident outliers.

**Table 1:** Definition and descriptive statistics of variables. SD is the standard deviation.

Variable name	Sign	Definition and explanation	Mean	SD	Min	Max
City carbon footprint pressure	CCF	Carbon emission equivalent/carbon sinks equivalent	1.363	1.824	0.009	16.51
Digital finance	DIF	Using data compiled by the “Peking University Digital Finance Research Center”	1.751	0.682	0.171	3.345
Degree of digitalization	DIG		1.659	0.671	0.018	3.265
Depth of use	USE		1.720	0.699	0.042	3.497
Breadth of coverage	COV		2.107	0.826	0.027	5.812
Economic development level	GDP	GDP/city population	0.053	0.034	0.006	0.468
Foreign trade dependence	FDI	City import and export trade volume/GDP	1.641	8.277	0.001	398
Educational foundation	EDU	Government education expenditure/total government financial expenditure	0.507	0.332	0.076	2.910
Technology level	ST	Government expenditure on science and technology/total government expenditure	0.016	0.017	0.001	0.207
Urbanization degree	URBAN	Non-agricultural population/(agricultural population + non-agricultural population)	0.497	0.208	0.116	2.355

### Model construction

To study the impact of digital finance on urban carbon footprint pressure, this study established the following estimation model:

3 $$ CCF_{i,t} = \alpha_{0} + \alpha_{1} DIF_{i,t} + \alpha_{2} Control_{i,t} + \gamma_{i} + \theta_{t} + \varepsilon_{i,t} $$

In formula (2), CCF_i,t_ represents the urban carbon footprint pressure, while DIF_i,t_ serves as the core explanatory variable for digital finance. Control_i,t_ comprises the control variables necessary for this study. *γ*_*i*_ denotes the fixed effects for individual cities, and *θ*_*t*_ encapsulates the fixed effects over time and years. Controlling for individual and temporal variations mitigates biases in the estimation results caused by individual differences and time trends. *ε*_*i,t*_ is the random disturbance. Here,* i* stands for the city, and *t* represents the year. Particular attention is given to the coefficient *α*_*1*_ of digital finance. If this coefficient is significantly negative, it suggests that digital finance effectively reduces the pressure of urban carbon footprints. Conversely, a positive coefficient would indicate that digital finance exacerbates this pressure. Additionally, to delve deeper into the intrinsic mechanism by which digital finance impacts urban carbon footprints, this study introduces interaction terms between the independent variables and mechanism variables based on Model (3), thereby constructing a mechanism testing model.

4 $$ CCF_{i,t} = \beta_{0} + \beta_{1} DIF_{i,t} + \beta_{2} DIF_{i,t} \times M_{i,t} + \beta_{3} Control_{i,t} + \gamma_{i} + \theta_{t} + \varepsilon_{i,t}  $$

In Formula (4), Mi,t represents the mechanism variable, which is the coefficient for the interaction term, with all other variables consistent with those in Model (3). Specifically, if the coefficient for digital finance is significantly negative, and the coefficient for the interaction term is also significantly negative, it indicates that digital finance can alleviate urban carbon footprint pressure through this specific mechanism. This suggests a synergistic effect where digital finance not only directly reduces carbon footprint pressures but also enhances the effect through the mechanism variable, thereby further contributing to sustainability in urban environments.

### Data validation

#### Differences in distribution of urban carbon footprint pressure

**Figure 2:**
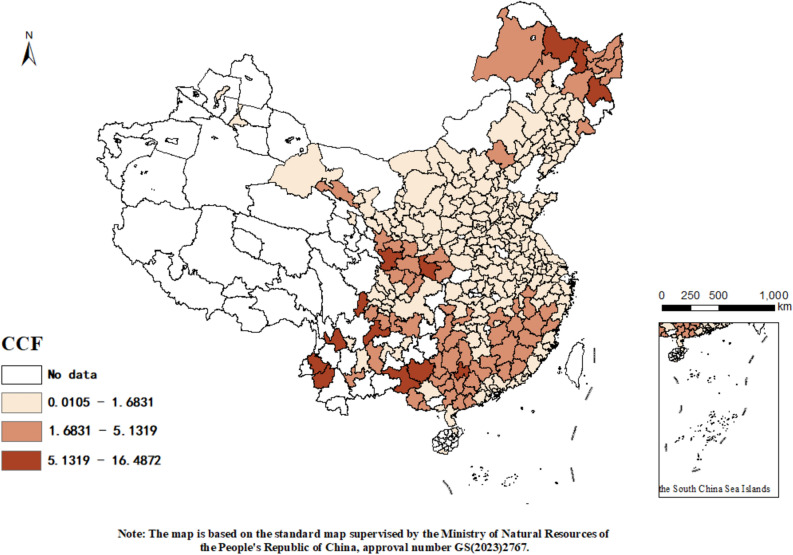
Distribution of city carbon footprint pressure in 2011.

**Figure 3:**
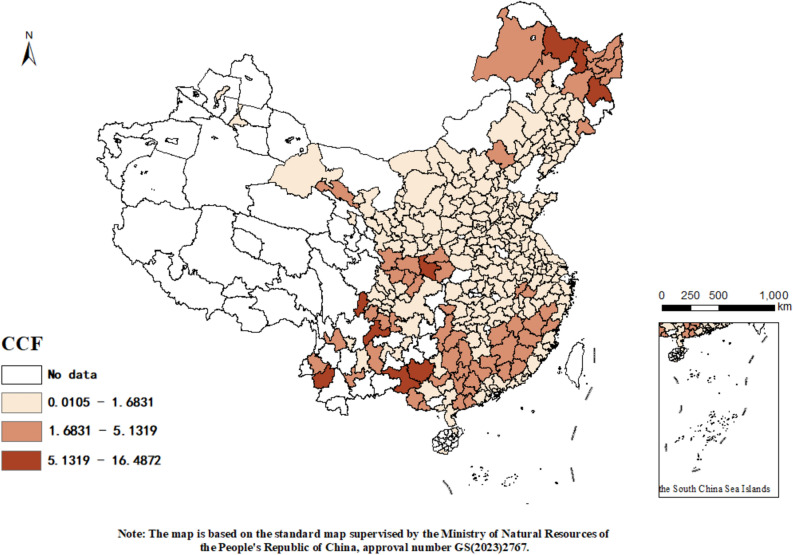
Distribution of city carbon footprint pressure in 2020.

To more effectively illustrate the spatial distribution of urban carbon footprint pressures, we utilized ArcGIS software to visualize the data from 2011 and 2020. Figures 2 and 3 respectively display the spatial distribution of carbon footprint pressures in Chinese cities for these years. Observations from these figures reveal a declining trend in urban carbon footprint pressures, indicating significant progress in emission reduction and carbon sinks efforts in recent years. Specifically, several key observations are noted:

##### Spatial differences

There are clear spatial disparities in the intensity of urban carbon footprint pressures. From 2011 to 2020, these disparities are evident across different regions. Central China shows minimal changes with most cities maintaining low levels of pressure (below 1.6831). In contrast, cities in Southern and Northern China exhibit significant fluctuations. Notably, some cities in the north have experienced an increase in carbon footprint pressures, likely due to the high energy demands for heating in winter and cooling in summer, typically relying on energy-intensive systems which further contribute to carbon emissions. Conversely, many cities in the south have shown a marked decrease, particularly along the middle and lower reaches of the Yangtze River. This north–south disparity may be attributed to the colder winters and hotter summers in the north, as compared to the rich forest resources in the south where efforts to protect and restore forests help absorb atmospheric CO_2_ and increase regional carbon sinks capacity, thus alleviating urban carbon footprint pressures.

##### Spatial clustering

The distribution of urban carbon footprint pressures exhibits clear spatial clustering. For instance, in the southern region, cities such as Nanping, Sanming, Longyan, and Meizhou show contiguous characteristics of carbon pressures. In central China, the carbon pressures are interconnected and generally lower. This clustering is likely due to the uneven distribution of industries across China, with some regions hosting large-scale manufacturing and heavy industries, while others focus more on services and light industries. The high carbon emissions associated with manufacturing activities thus lead to clustered patterns in carbon footprint pressures.

This analysis not only highlights the regional variations and trends in carbon footprint pressures but also emphasizes the influence of industrial distribution and environmental policies on urban carbon management.

#### Trends in urban carbon footprint pressures

**Figure 4:**
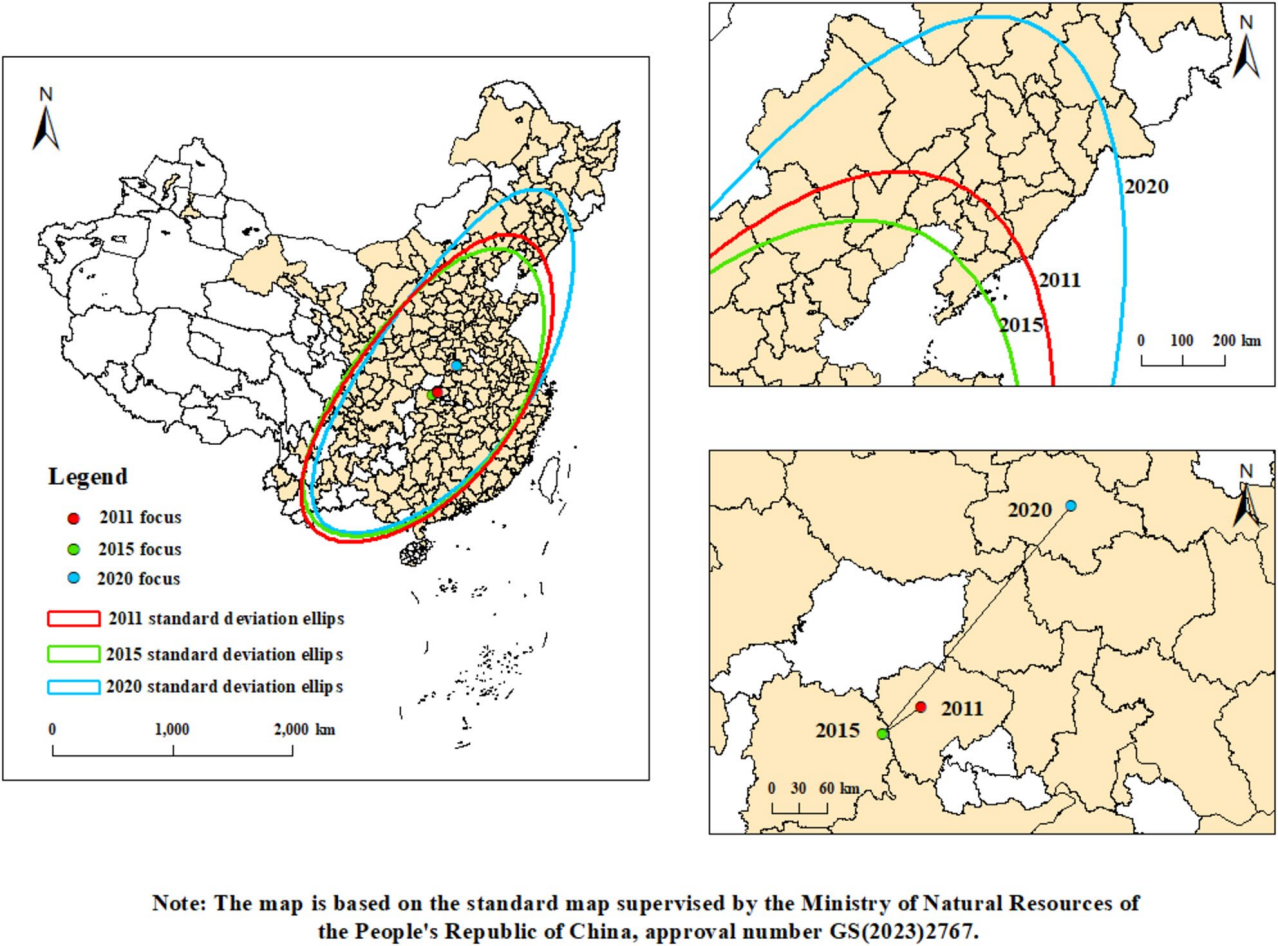
Standard deviation elliptical distribution and center of gravity migration trajectory chart.

To analyze the trends in the distribution and centroid migration of urban carbon footprints, this study employed standard deviation ellipses in its mapping analysis. Figure 4 illustrates the ellipses for urban carbon footprints pressures in 2011, 2015, and 2020, along with their centroid migration trajectories. Based on the observations from Fig. 4, we can draw several conclusions:

Firstly, the spatial distribution of urban carbon footprint pressures in China for the years 2011, 2015, and 2020 predominantly followed a "northeast-southwest" orientation. This pattern is consistent with the trajectory of the Hu Line, indicating that urban carbon footprint pressures align with levels of economic development and population distribution.

Secondly, there was a noticeable trend of decreasing minor axes and increasing major axes of the standard deviation ellipses as the years progressed. This shift from a concentrated to a polarized spatial distribution of urban carbon footprint pressures highlights the stark regional differences in these pressures.Thirdly, further analysis reveals that between 2011 and 2020, the migration route of the urban carbon footprint pressure centroid initially moved towards the southwest before shifting to the northeast. This shift was largely driven by policies introduced by the Chinese government to reduce environmental pollution in the eastern regions. These policies led to the relocation of secondary industries to the southwest, thereby intensifying urban carbon footprint pressures in that region. Moreover, from 2015 to 2020, the centroid of urban carbon footprint pressures moved towards the northeast, likely due to the presence of heavy industries and manufacturing sectors in the northern regions, which are typically associated with high carbon emissions. Additionally, these industries often face significant challenges in achieving green transformation, complicating efforts towards carbon neutrality and exacerbating urban carbon footprint issues.

These findings confirm that urban carbon footprint pressure is driven by factors such as economic development and population activity. Furthermore, they underline the importance of using financial instruments to achieve coordinated development of the economy and the environment in today's era. This emphasizes the need for digital financial developments that not only promote economic growth but also ensure environmental sustainability, thus addressing the twin challenges of economic and ecological health in urban environments.

### Empirical analysis

#### Basic estimation model

Table 2 presents the baseline regression results, where the dependent variable is urban carbon footprint pressure. Columns (1) and (2) utilize a two-way fixed effects model for regression. To reduce bias from model selection, columns (3) and (4) adopt a random effects model, assuming that all individuals share the same intercept, and differences between individuals are random, primarily reflected in the settings of random disturbance terms. Additionally, columns (1) and (3) of Table 2 do not include control variables, while columns (2) and (4) incorporate them.Observing the baseline regression results, under the fixed effects model, the coefficient of digital finance is significantly negative at the 1% level, with values of − 0.4112 and − 0.3455 when control variables are not included and included, respectively. This indicates that digital finance can effectively reduce the pressure of urban carbon footprints, thus confirming Hypothesis 1. Furthermore, under the random effects model, the coefficient of digital finance remains significantly negative at the same 1% level. This result strengthens the evidence of digital finance's role in mitigating urban carbon footprint pressures.

**Table 2:** Benchmark regression results.

	Fixed effects model		Random effects model	
	(1)	(2)	(3)	(4)
DIF	− 0.4112*** (− 3.44)	− 0.3455*** (− 2.69)	− 0.5775*** (− 4.92)	− 0.5236*** (− 4.11)
GDP		− 0.0321 (− 0.52)		− 0.0715 (− 1.14)
FDI		− 0.0002 (− 0.31)		− 0.0003 (− 0.37)
EDU		− 0.1905*** (− 2.68)		− 0.0572 (− 0.81)
ST		− 0.6001 (− 0.71)		− 1.0005 (− 1.17)
URBAN		0.4749*** (2.77)		0.0945 (0.58)
Constant	− 1.5983*** (− 24.32)	1.4401*** (11.40)	1.6850*** (14.20)	1.6807*** (10.98)
City FE	Yes	Yes	Yes	Yes
Year FE	Yes	Yes	Yes	Yes
R^2^	0.1340	0.0775	0.1406	0.1132
N	2770	2770	2770	2770

The "t" value is represented in parentheses. *** represents that the estimated coefficient is significant at the 1%, level.

### Multi-dimensional analysis of digital finance

To delve deeper into the impact of various developmental dimensions of digital finance on urban carbon footprint pressures, secondary indicators of the digital finance index—coverage rate, usage depth, and degree of digitalization—were used as independent variables for estimation and testing. Table 3 reports the test results. Notably, the coefficient for digital finance coverage rate is significantly negative at the 1% level, with a value of − 0.9385, indicating that an expanded coverage of digital finance effectively suppresses urban carbon footprint pressures. However, it is important to note that the usage depth and degree of digitalization reported in columns (2) and (3) of Table 3 did not pass the significance tests. This result suggests that the insufficient depth of use and level of digitalization are key obstacles in the role of digital finance in reducing urban carbon footprint pressures. Several reasons may account for these outcomes:

**Table 3:** Multidimensional regression results.

	(1) CCF	(2) CCF	(3) CCF
COV	− 0.9385*** (− 7.02)		
USE		0.1189 (1.41)	
DIG			0.0132 (1.41)
Constant	− 1.7220*** (− 7.02)	1.1651*** (10.05)	1.2322*** (11.72)
Control	Yes	Yes	Yes
City FE	Yes	Yes	Yes
Year FE	Yes	Yes	Yes
R^2^	0.0478	0.1389	0.1370
N	2770	2770	2770

The "t" value is represented in parentheses.*** represents that the estimated coefficient is significant at the 1%, level.

First, a significant portion of the population lacks understanding and awareness of digital finance products and services. Many urban residents, particularly those less familiar with digital technologies, face challenges in understanding the operation, benefits, and potential risks of digital finance. This lack of understanding constitutes both a psychological and practical barrier that hinders the adoption and optimal use of digital finance services, thereby restraining their role in mitigating carbon footprint pressures. Second, issues related to personal data security and privacy dampen users' trust in digital finance, hindering their engagement with these services. This problem is exacerbated by notable cyber attacks and insufficient regulatory frameworks, failing to instill confidence in potential users. This phenomenon further impedes the alleviating impact of deep usage of digital finance on urban carbon footprint pressures. Lastly, many urban areas, especially among low-income groups, still have limited access to digital technologies. The digital divide encompasses not only access issues but also variations in connection quality and the affordability of digital tools. These limitations restrict a significant portion of the urban population from participating in the digital finance ecosystem, thus limiting its potential to mitigate carbon footprint pressures effectively.

### Robustness analysis

1. *Instrumental variable method.* In the baseline regression, even though time variations and individual differences are controlled, the potential correlation between the independent variables and the error terms still results in endogeneity issues. Therefore, this study adopts the instrumental variable (IV) method to decompose the endogenous variable into endogenous and exogenous components to eliminate possible endogenous effects. Building on the research by Li^18^, this study employs the Internet penetration rate as an instrumental variable. Columns (1) and (2) of Table 4 report the results of the two-stage least squares (2SLS) instrumental variable method, where column (1) shows the first-stage regression results and column (2) shows the second-stage regression results in detail. In addition, the instrumental variable (Inter) passed the Wald F-value test and the weak instrumental variable test, confirming that Internet penetration rate is an effective instrumental variable. The regression results show that even after further eliminating the endogenous effects, digital finance still has a suppressive effect on urban carbon footprint pressure.
2. *Exclusion of special samples method.* We recognize that centrally administered municipalities typically possess more fiscal resources and national support compared to other towns and cities. These advantages often enable such municipalities to develop more rapidly in economic and educational terms. To mitigate the impact of these factors, this paper re-evaluates the data excluding samples from centrally administered municipalities and performs linear estimation again. Column (3) of Table 4 presents the results after the exclusion of these municipalities. The coefficient of digital finance remains significantly negative at the 1% level, indicating that digital finance continues to alleviate urban carbon footprint pressures even when excluding the special influences of municipal authorities.
3. *Eliminate bias in pilot cities.* Pilot cities for carbon emission reduction are subject to more stringent policies and regulations aimed at decreasing emissions, coupled with policies that encourage such reductions. These factors could potentially confound the positive effects of digital finance. To avoid the influence of pilot cities and to enhance the generality and purity of the sample characteristics, this study excludes cities identified as carbon emission pilot cities during the survey period in its robustness tests. Specifically, data from 11 cities over five years were excluded, resulting in 55 observational samples. Column (4) of Table 4 presents the regression results after the exclusion of pilot cities. The coefficient for digital finance remains significantly negative, indicating that the baseline regression remains robust after removing the effects of the pilot cities.
4. *Evaluating the impact of external policies on digital finance.* To assess the influence of external policies or events on digital finance and thereby isolate the effects of policy from those due to endogeneity issues, consider the 2016 initiative under the "G20 High-Level Principles on Digital Inclusive Finance," where China advocated the use of digital technologies to promote inclusive finance. This policy greatly accelerated the development of digital finance. However, the establishment of financial service infrastructures has also introduced new carbon emission challenges. Thus, the question arises: does this policy impact the effectiveness of digital finance? Column (5) of Table 4 shows the test results for exogenous policy impact. Since the policy was implemented in 2016, the period from 2016 to 2020 serves as the regression test window. The results indicate that the coefficient of digital finance is significantly negative at the 1% level, suggesting that digital finance continues to mitigate urban carbon footprint pressures even under the influence of this policy.
5. *Validation using firm-level data in baseline regression.* In our baseline regression, we utilized city-level data for analysis. To enhance the robustness of our research findings, we now validate these results using firm-level data. First, we assess the level of digital finance in firms based on the development of digital finance in their respective registered locations. We then substitute firm-level carbon emissions for urban carbon footprint pressures, as carbon sinks is typically measured at the macro level and cannot be precisely quantified by firms. Table 4, Column (6) displays the regression results at the firm level. The results indicate that the coefficient of digital finance is significantly negative at the 1% level, demonstrating that the integration of digital finance effectively reduces carbon emissions in firm production and operations. This suggests that the application of digital finance at the firm level can alleviate carbon footprint pressures, further confirming the results of the baseline regression.
6. *Adding control variables.* Considering that existing control variables already address aspects of urban economy, education, technology, and urbanization, there could still be biases due to omitted variables, especially those related to urban carbon footprints. To address this, this study introduces additional control variables: per capita green space and per capita park area. As primary sources of urban carbon sinks, green spaces and parks play a significant role in the urban carbon cycle and climate change mitigation. Incorporating these variables aims to more directly mitigate the omission of relevant environmental features. Table 5, Column (1), reports the regression results with these characteristic control variables added. The coefficient of digital finance is significantly positive, indicating that the baseline regression results remain valid even when controlling for environmental characteristics.
7. *Variable measurement substitution method.* Different measurement methods can introduce varying degrees of error and uncertainty. Substituting the measurement approaches for both independent and dependent variables can help reduce these impacts. The foundation of digital finance is the digital transformation of financial services, where finance essentially facilitates the conversion of savings into investments by individuals and households, allows enterprises to raise funds for expansion, and enables governments to fund public projects. Thus, this study substitutes the level of financial development for digital finance, measuring urban financial development using the logarithm of city residents' savings and financial management. Furthermore, since the root cause of urban carbon footprint pressure is excessive carbon emissions, Carbon Dioxide Equivalent (CDE) is used as a substitute for urban carbon footprint pressure as the dependent variable. Columns (2) and (3) of Table 5 display the test results using the substituted variable measurement methods. The results indicate that the substituted variables still pass the significance tests, further enhancing the robustness of the baseline regression results.

**Table 4:** Instrumental variable method and replacement sample test results.

	2SLS		Eliminate municipalities	Eliminate pilot cities	Exogenous policy shock	Enterprise level	Add feature variable	Replace independent variable	Replace dependent variable
	(1) DIF	(2) CCF	(3) CCF	(4) CCF	(5) CCF	(6) CCF	(7) CCF	(8) CDE	(9) CCF
DIF		− 0.2868* (− 1.82)	− 0.3544*** (− 2.72)	− 0.3458*** (− 2.59)	− 0.8851*** (− 2.65)	− 1.097*** (− 3.59)	− 0.3748*** (− 2.86)		− 1.5674*** (− 3.63)
Inter	0.0035*** (2.88)								
Finance								− 0.8627*** (− 4.37)	6.9301*** (− 16.31)
Constant	1.3101*** (18.93)	0.4476* (1.70)	1.8891*** (7.48)	1.8839*** (7.30)	2.9798*** (3.42)	1.4671*** (4.12)	1.5417*** (9.53)	7.1761*** (5.26)	6.9301*** (− 16.31)
Control	Yes	Yes	Yes	Yes	Yes	Yes	Yes	Yes	Yes
City FE	Yes	Yes	Yes	Yes	Yes	Yes	Yes	Yes	Yes
Year FE	Yes	Yes	Yes	Yes	Yes	Yes	Yes	Yes	Yes
R^2^	0.2352	0.2335	0.0117	0.0116	0.0159	0.0159	0.1104	0.0625	0.0884
N	2770	2770	2730	2675	1385	14,705	2770	2770	2770

The "t" value is represented in parentheses. *** and * represent that the estimated coefficient is significant at 1% and 10% levels, respectively.

**Table 5:** Mechanism test results.

	(1) CCF	(2) CCF
DIF	− 0.8059*** (− 4.97)	− 0.3111** (− 2.40)
DIF*BANK	0.5396*** (4.62)	
DIF*PEC		− 0.0003* (− 1.90)
Constant	1.4283*** (11.35)	0.7501 (10.05)
Control	Yes	Yes
City FE	Yes	Yes
Year FE	Yes	Yes
R^2^	0.1319	0.1568
N	2770	2770

The "t" value is represented in parentheses. ***, **, and * represent that the estimated coefficient is significant at the 1%, 5%, and 10% levels, respectively.

### Influence path analysis

1. *Reducing the number of bank branches.* The determinants of the number of bank branches in cities are highly complex, influenced by various factors such as market conditions, economic conditions, technology, and policies^36^. Therefore, this study selects the number of bank branches (Bank) as one of the mechanism variables and measures it by the number of bank branches in the city. Additionally, we use Model (2) to test this mechanism, with the estimated results shown in column (1) of Table 5. The interaction term between digital finance and the number of bank branches is significantly positive at the 1% level, while the coefficient for digital finance is significantly negative at the 1% level.This result indicates that digital finance can play an important role in mitigating urban carbon footprint pressure by reducing the number of bank branches in the city. Hypothesis 2 is thus confirmed.
2. *Increasing residents' environmental awareness.* With the iterative upgrading of digital technology, the internet can use big data to timely capture residents' attention to environmental causes, reflecting their preferences and intentions towards natural resource conservation or green living. Moreover, residents' attention to the environment can effectively reduce the likelihood of polluting enterprises entering the market, thus controlling pollution emissions at the source^37^. To test Hypothesis 3, this study quantifies residents' environmental awareness (PEC). We use Baidu search engine queries for environmental keywords to measure residents' environmental awareness. This method has been widely applied in existing research^38,39^. Column (2) of Table 5 reports the potential mechanism of residents' environmental awareness. The coefficient for digital finance is significantly negative at the 1% level, and the coefficient for the interaction term is also significantly negative, indicating that the application of digital finance has increased residents' environmental awareness, thereby playing an important role in reducing urban carbon footprint pressure. Hypothesis 3 is thus confirmed.

### Nonlinear analysis

Further reflection reveals that the development of digital finance relies on the iterative advancement of digital technology, which, as a form of production technology innovation, exhibits marginal increasing returns. In the early stages of technological innovation, the learning curve effect typically emerges. In simple terms, as the frequency of use increases and experience accumulates, production efficiency and technological application capabilities gradually improve. Initial investments and learning costs are high, but over time, these costs decrease, making the application of technology more efficient^40^. These marginal changes subsequently affect digital finance, potentially resulting in diminishing returns. Beyond technological factors, the nonlinear effects of digital finance are also evident in the snowball effect of data collection and retrieval, where data accumulation may lead to marginal increasing returns. Consequently, due to technological or market factors, the impact of digital finance on urban carbon footprints may also be nonlinear.Based on this, our study incorporates a quadratic term for digital finance into the original Model (1) to capture the nonlinear relationship, aiming to further understand the complex relationship between digital finance and urban carbon footprint pressure. If the coefficient of the quadratic term passes the significance test, it indicates the presence of nonlinear effects.

Table 6 reports the results of the nonlinear effect test. The coefficient for the linear term of digital finance is significantly negative at the 1% level, while the coefficient for the quadratic term of digital finance is significantly positive at the 1% level. This result indicates that digital finance exerts a nonlinear effect on urban carbon footprint pressure. To further verify the nature of this nonlinear relationship, this study calculates the nonlinear inflection point. The calculation reveals that the nonlinear inflection point is 3.9, whereas the maximum value of digital finance is approximately 3.3. This result suggests that the impact of digital finance follows a linear relationship and shows a monotonic change within the investigated range. However, when the level of digital finance exceeds the inflection point, a nonlinear relationship emerges.

**Table 6:** Nonlinear test.

	(1) CCF
DIF	− 0.8791*** (− 4.45)
DIF^2^	0.1132*** (3.55)
Constant	1.6055*** (11.95)
Control	Yes
City FE	Yes
Year FE	Yes
R^2^	0.0431
N	2770

The "t" value is represented in parentheses. *** represents that the estimated coefficient is significant at the 1%, level.

**Figure 5:**
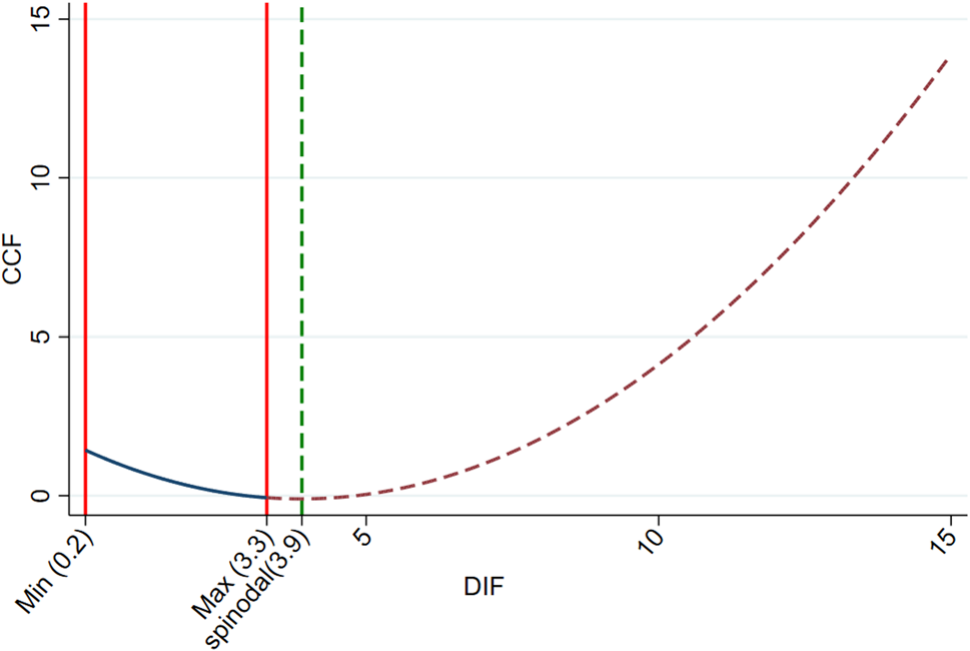
Nonlinear change trend chart.

To visually illustrate this change, the study visualizes the nonlinear relationship. Figure 5 shows the visualized nonlinear variation. Within the investigated range of digital finance, as digital finance increases, the pressure on urban carbon footprints gradually decreases. However, the suppression rate gradually diminishes according to the curve's variation amplitude. This phenomenon could be because the deeper development of digital finance leads urban residents to rely more on electronic devices such as smartphones, tablets, and computers, resulting in a sharp increase in electricity consumption, which is detrimental to alleviating urban carbon footprint pressure. Therefore, it is necessary to seek a balance between the efficiency brought by digital finance and its negative impacts to achieve long-term green and low-carbon development.

### Heterogeneity analysis

#### Regional differences

Considering the potential differences in natural and human factors across various locations is crucial. Natural factors include climate, natural resources, topographical features, and ecosystem conditions, while human factors encompass economic disparities, cultural customs, urbanization differences, and policy variations. These differences can impact the influence of digital finance on urban carbon footprint pressure in different ways. This study examines the inhibitory effect of digital finance on urban carbon footprint pressure in different regional contexts. Table 7 reports the sub-sample regression results for China's three major regions. From the regression results, it is evident that the inhibitory effect of digital finance on urban carbon footprint pressure is most significant in the central region, followed by the eastern region, while the effect in the western region is not significant. The possible reason for this is that compared to the eastern and central regions, the western region lags economically, with relatively lower levels of urban infrastructure, technology, and digital maturity. The application of digital finance technology is limited in this area. This limitation hinders the full potential of digital finance in energy management, resource allocation, and carbon footprint reduction. Consequently, the alleviation effect on urban carbon footprint pressure is not evident in the western region.

**Table 7:** Regional heterogeneity.

	East area	Central area	Western area
	(1) CCF	(2) CCF	(3) CCF
DIF	− 0.2014** (− 2.47)	− 1.9741*** (− 5.94)	0.1458 (0.83)
Constant	0.9804*** (5.76)	4.3914*** (7.64)	1.5111*** (3.83)
Control	Yes	Yes	Yes
City FE	Yes	Yes	Yes
Year FE	Yes	Yes	Yes
R^2^	0.0218	0.0777	0.0184
N	1000	1040	730

The "t" value is represented in parentheses. *** and ** represent that the estimated coefficient is significant at 1% and 5% levels, respectively.

#### Differences in sunlight duration

The primary source of carbon sinks in urban areas is vegetation, which absorbs atmospheric carbon dioxide through photosynthesis and stores it as organic matter. The main driver of photosynthesis is solar radiation; therefore, sunlight duration is a key factor influencing the carbon sinks capacity of urban vegetation. This study considers that due to the significant latitudinal differences between northern and southern China, the solar altitude angle varies greatly among cities, resulting in different sunlight durations. Additionally, diverse topographies, such as mountainous areas and plateaus, create shadow effects, leading to shorter sunlight durations in some cities, while sunlight duration is relatively longer in foothill or plain areas.Based on these factors, we explore the differential effects of digital finance on alleviating urban carbon balance pressure under varying sunlight durations. It is important to note that urban sunlight duration is calculated based on the interval between sunrise and sunset. Sunrise is defined as the moment when the solar altitude angle exceeds 0 degrees, and sunset is defined as the moment when it falls below 0 degrees. The solar altitude angle is determined by the city's dimensions, solar declination, and solar hour angle. Table 8 reports the regression results for samples with long and short sunlight durations. We use the 50th percentile of all samples as the critical value. Samples exceeding this critical value are categorized as having long sunlight durations, while those below it are categorized as having short sunlight durations. In the regression for long sunlight duration samples, digital finance significantly alleviates urban carbon footprint pressure. However, in the regression for short sunlight duration samples, digital finance increases urban carbon footprint pressure, indicating that shorter sunlight durations are unfavorable for reducing carbon footprint pressure.

**Table 8:** Daylight duration heterogeneity.

	Long daylight duration	Short daylight duration
	(1) CCF	(2) CCF
DIF	− 0.6833** (− 2.54)	0.4447*** (3.96)
Constant	1.1686* (1.93)	0.8622*** (4.05)
Control	Yes	Yes
City FE	Yes	Yes
Year FE	Yes	Yes
R^2^	0.0358	0.0277
N	1246	1488

The "t" value is represented in parentheses. ***, **, and * represent that the estimated coefficient is significant at the 1%, 5%, and 10% levels, respectively.

## Conclusions and discussions

### Conclusions

This article explores the role of digital finance in promoting sustainable urban development, particularly in mitigating climate change and promoting economic growth. Using data from 277 cities in China from 2011 to 2020, we evaluated how digital finance affects urban carbon footprint pressure. Different from previous studies, this study highlights three key findings: First, we identify the spatial variation of China's urban carbon footprint and combine it with economic development trends, emphasizing the key role of economic models in shaping urban carbon footprints. Secondly, this study verifies that the development of digital finance can effectively reduce urban carbon footprint pressure. This provides strong empirical support for digital finance’s contribution to sustainable development. Finally, our research shows that digital finance alleviates carbon footprint pressure by reducing the number of physical bank branches and increasing residents’ environmental awareness. Meanwhile, heterogeneity analysis shows that this mitigating effect is more pronounced in eastern and central China and in cities with longer sunshine hours. These insights not only highlight the potential of digital finance to reduce cities’ carbon footprints, but also provide valuable guidance to policymakers, particularly in developing strategies that take into account regional differences and economic policies.

### Lessons learnt

Firstly, given digital finance's significant role in mitigating urban carbon footprint pressures and its variable regional impacts, governments should develop tailored digital finance policies aligned with specific local economic and environmental conditions. It is crucial to promote digital finance in regions with high carbon emissions and in cities experiencing extensive sunlight. Such targeted policies would not only leverage digital technology to foster economic and environmental synergy but also optimize resource allocation. Secondly, it is advisable for governments to enhance investments in digital infrastructure and offer incentives for innovation, such as tax breaks and research funding, to bolster fintech companies and other innovative entities. Strengthening infrastructure and technological innovation will not only broaden the reach and efficacy of digital financial services but also amplify their role in diminishing urban carbon footprints, thereby supporting sustainable urban development. Lastly, governments should utilize digital platforms to extensively disseminate environmental knowledge and boost residents' engagement with environmental issues. Encouraging the development and promotion of digital financial applications that incorporate environmental data monitoring and analysis, like offering eco-friendly rewards and carbon credit scores through mobile banking apps, can integrate advancements in digital technology with improvements in residents' environmental conduct, thus advancing environmental protection objectives.

### Limitations and future work

Due to the extensive period and broad coverage of macro data, it offers valuable insights for policy development and forecasting. Consequently, this study utilizes macro-city data to investigate the mitigating effects of digital finance on carbon footprint pressure. Nonetheless, it is essential to recognize that microdata more precisely reflects the specific nuances of distinct groups. Given the limitations in accessing data on financial literacy and personal carbon emissions, we are unable to evaluate the impact of digital finance at the micro-individual level in reducing carbon footprint pressures. Future research will employ household questionnaires to gather data on micro-individual digital financial literacy and carbon footprints, thereby expanding the research scope and providing a more comprehensive and robust basis for policymakers.

## Data Availability

Data related to the paper can be obtained from the corresponding author upon request.
